# Artificial Intelligence-Based Sentinel Lymph Node Metastasis Detection in Cervical Cancer †

**DOI:** 10.3390/cancers16213619

**Published:** 2024-10-26

**Authors:** Ilse G. T. Baeten, Jacob P. Hoogendam, Nikolas Stathonikos, Cornelis G. Gerestein, Geertruida N. Jonges, Paul J. van Diest, Ronald P. Zweemer

**Affiliations:** 1 Department of Gynecologic Oncology, Division of Imaging and Oncology, University Medical Center Utrecht, Utrecht University, 3584 CX Utrecht, The Netherlandsr.zweemer@umcutrecht.nl (R.P.Z.); 2 Department of Pathology, University Medical Center Utrecht, Utrecht University, 3584 CX Utrecht, The Netherlands

**Keywords:** deep learning, artificial intelligence, ultrastaging, sentinel lymph node, cervical cancer

## Abstract

**Background/objectives**: Pathological ultrastaging, an essential part of sentinel lymph node (SLN) mapping, involves serial sectioning and immunohistochemical (IHC) staining in order to reliably detect clinically relevant metastases. However, ultrastaging is labor-intensive, time-consuming, and costly. Deep learning algorithms offer a potential solution by assisting pathologists in efficiently assessing serial sections for metastases, reducing workload and costs while enhancing accuracy. This proof-of-principle study evaluated the effectiveness of a deep learning algorithm for SLN metastasis detection in early-stage cervical cancer. **Methods**: We retrospectively analyzed whole slide images (WSIs) of hematoxylin and eosin (H&E)-stained SLNs from early-stage cervical cancer patients diagnosed with an SLN metastasis with either H&E or IHC. A CE-IVD certified commercially available deep learning algorithm, initially developed for detection of breast and colon cancer lymph node metastases, was employed off-label to assess its sensitivity in cervical cancer. **Results**: This study included 21 patients with early-stage cervical cancer, comprising 15 with squamous cell carcinoma, five with adenocarcinoma, and one with clear cell carcinoma. Among these patients, 10 had macrometastases and 11 had micrometastases in at least one SLN. The algorithm was applied to evaluate H&E WSIs of 47 SLN specimens, including 22 that were negative for metastasis, 13 with macrometastases, and 12 with micrometastases in the H&E slides. The algorithm detected all H&E macro- and micrometastases with 100% sensitivity. **Conclusions**: This proof-of-principle study demonstrated high sensitivity of a deep learning algorithm for detection of clinically relevant SLN metastasis in early-stage cervical cancer, despite being originally developed for adenocarcinomas of the breast and colon. Our findings highlight the potential of leveraging an existing algorithm for use in cervical cancer, warranting further prospective validation in a larger population.

## Simple Summary

Currently, pathologists use ultrastaging to detect whether cancer has spread to the lymph nodes. This process is time-consuming and expensive. Our pilot study explored the use of a deep learning algorithm to help detect cancer spread to lymph nodes of early-stage cervical cancer patients. Using this technology could make the detection process faster, more efficient, and less costly. We evaluated an algorithm that was originally designed to identify cancer spread to lymph nodes in breast and colon cancer in cervical cancer patients. The study included 21 women with different types of early-stage cervical cancer. The algorithm was used to analyze 47 lymph node samples and successfully identified all cases where cancer had spread, showing 100% accuracy. Although the algorithm was initially developed for other cancers, it proved highly effective in this new population. More prospective research in a larger group of patients is needed to confirm its cost-effectiveness.

## 1. Introduction

Lymph node involvement is the most important prognostic factor in early-stage cervical cancer and influences therapy decisions [1]. To assess nodal involvement, sentinel lymph node (SLN) biopsy and subsequent pathological processing according to the ultrastaging protocol is recommended in early-stage cervical cancer [2]. Ultrastaging, consisting of serial step sectioning at multiple levels and immunohistochemical staining, is accurate for detecting low volume disease, defined as either micrometastasis or isolated tumor cells, but is a labor-intensive and expensive procedure [3,4,5]. In addition, with a risk of around 18% for nodal involvement in this population, only about 20% of the metastases are exclusively detected by ultrastaging [6]. With a median of two SLNs per patient, numerous SLNs must be processed for one additional finding of metastasis [7]. Consequently, the cost-effectiveness of ultrastaging is still debated and leaves room for other technologies.

Recently, artificial intelligence (AI)-based algorithms have been developed offering automated detection of metastases in whole slide images (WSIs) of SLNs. This technology potentially reduces the pathologists’ workload while increasing efficiency, diagnostic accuracy, and reproducibility [8]. Furthermore, an algorithm aiding the pathologist in screening the hematoxylin and eosin (H&E) sections may reduce the need for immunohistochemistry (IHC) and could benefit the cost-effectiveness of ultrastaging. AI algorithms for detection of nodal metastases in breast cancer have shown to be comparable, or even superior, to a panel of pathologists in a simulated environment [9,10]. In a real-life clinical setting, the best diagnostic accuracy and highest efficiency may be reached when pathologists are assisted by AI-based algorithms [11,12]. Also, a recent clinical trial in breast cancer showed that the use of immunohistochemistry and subsequent SLN processing costs were significantly reduced for AI-assisted pathologists [13].

To the best of our knowledge, a deep learning algorithm for detecting nodal metastases in cervical cancer has not yet been developed. For creation and validation of new algorithms, large training and validation sets are needed. Although the histological characteristics may differ, as the majority of the cervical cancers are of squamous cell origin, the certified algorithms for breast cancer might also adequately detect nodal metastases in cervical cancer since they seem to detect anything not belonging in lymph nodes, potentially saving significant cost and time [13].

In this proof-of-principle study, we aimed to retrospectively assess the standalone performance of an existing deep learning application, certified to detect SLN metastases of breast and colon adenocarcinomas, for the detection of SLN metastases in early-stage cervical cancer. Our main objectives were to assess its sensitivity and to evaluate if this algorithm has the potential to be used outside the certified tumor range.

## 2. Materials and Methods

Patients were identified from a database with histologically proven cervical cancer patients who underwent primary surgery, including SLN procedure. This study included the early-stage cervical cancer patients diagnosed with SLN metastases, either macrometastasis (>2 mm) or micrometastasis (>0.2 mm and ≤2 mm) detected with either H&E or IHC, upon surgical lymph node staging from January 2015 to October 2023. Of these patients, we retrospectively collected all WSIs of H&E-stained SLN specimens to be reviewed by the algorithm. Excluded were patients with a finding of isolated tumor cells only, as the clinical value of this finding is disputed, or patients who objected against the reuse of their health care data for scientific research (as reported in their medical records). By including only patients with at least one positive SLN, we eliminated the risk of ambiguity if a case previously assessed as negative for metastasis would now be assessed as positive by the algorithm-supported pathologist. By selecting this subset of patients, no risk of an inferior diagnosis (i.e., missed tumor cells at prior diagnosis) existed. Ethics approval was waived for this retrospective cohort study. In this work, the study presented in [14] is revised and expanded.

All excised SLN specimens were routinely processed according to the ultrastaging protocol as previously described [7]. In 2015, we implemented a fully digital diagnostic workflow in which all slides were digitized as WSIs by Hamamatsu XR Nanozoomer 2.0 and S360 scanners (Hamamatsu Photonics, Hamamatsu City, Japan) at 40× magnification and reviewed using Sectra’s Picture Archiving and Communication System (PACS, Sectra AB, Linköping, Sweden) [15]. The included SLNs were categorized as negative, micrometastasis, or macrometastasis based on the largest tumor deposit identified at diagnosis by either H&E or IHC, as part of the clinical standard of care. This upfront diagnosis was considered the reference standard. If the area of metastasis had not yet been annotated by a pathologist at the WSI level at diagnosis, this was performed retrospectively by a pathologist (P.J.v.D.). Samples with metastases detected during the frozen section procedure were also included in this study.

**Figure 1:**
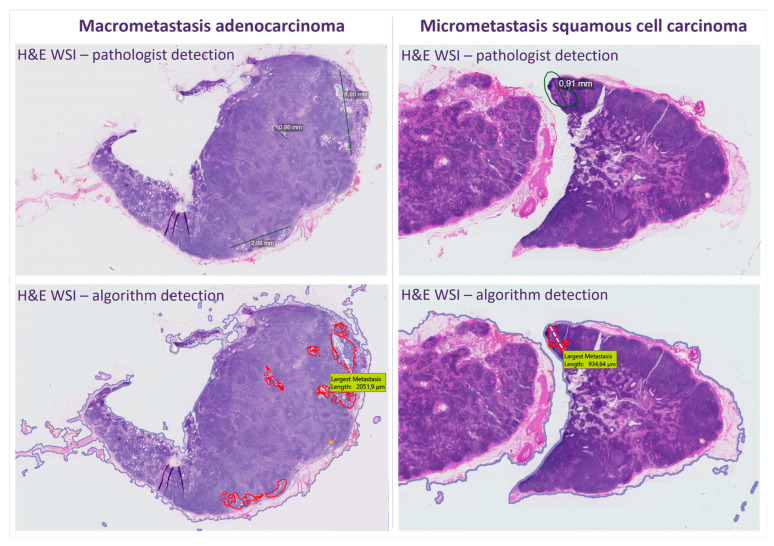
Output of a deep learning algorithm applied to H&E WSIs of sentinel lymph nodes in early-stage cervical adenocarcinoma and squamous cell carcinoma with correct detection of macrometastasis (**left**) and micrometastasis (**right**). H&E, hematoxylin and eosin; WSIs, whole slide images. *This figure was constructed from original output from PACS and Visiopharm. As these applications are set to European standards, the comma is used as decimal separator*.

All H&E WSIs were reviewed by the Conformité Européenne-in vitro diagnostics (CE-IVD)-certified Metastasis Detection App (version 2023.01.09) by Visiopharm (Hoersholm, Denmark), a deep-learning application developed to detect lymph node metastases from adenocarcinomas of breast cancer and colon cancer. This application was integrated within Sectra PACS where the output of the algorithm was graphically displayed. Details on the workflow of the app have been described before [12,13]. In summary, the Metastasis Detection App by Visiopharm marks suspicious cells with either red (high suspicion), orange (intermediate suspicion), or yellow (low suspicion) outlines. According to Visiopharm, the probability of a region being metastasis is based on the probability distribution generated by the neural network’s softmax function. The application provides results at the 95%, 80%, and 50% operating points, corresponding to red, orange, and yellow outlines. The best balance between sensitivity and specificity occurs at the 95% operating point (red outlines). The largest area of metastasis is indicated in mm^2^ and the largest length in mm (also see Figure 1). In this study, all outlined areas (either red, orange, or yellow) were considered equal as the most important aspect within an AI-assisted workflow is that the pathologist’s attention is drawn to the tumor area, regardless of the level of suspicion.

**Figure 2:**
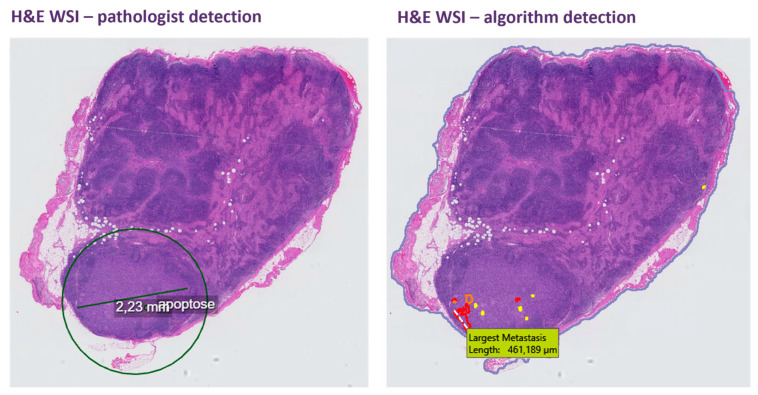
Example of algorithm output with multiple annotations of different colors in the correct area but not with the correct diameter. H&E, hematoxylin and eosin; WSI, whole slide image. *This figure was constructed from original output from PACS (with the original pathologist’s annotations) and Visiopharm. As these applications are set to European standards, the comma is used as decimal separator. Apoptose means apoptosis*.

The primary outcome was the standalone performance of the algorithm for detecting nodal metastases. Assessing performance consisted of checking whether the confirmed areas of metastases (by a pathologist), either on the H&E slides or IHC slides, were annotated by the algorithm on the H&E slides, covering the same area either partially or entirely, as the key parameter was that the pathologist’s review should be guided to this area (see Figure 2). This outcome was made binary: metastases were either correctly annotated (regardless of color and diameter), or they were not. The annotations by the algorithm were reviewed by a researcher (I.G.T.B.) and pathologist (P.J.v.D.) and compared to the reference annotations made by pathologists at diagnosis. In case of an apparent false-negative finding with the algorithm, a second pathologist reviewed the slides (G.N.J.). The standalone performance of the algorithm was expressed by its sensitivity for detection of SLN metastases: true-positive SLNs/(true-positive SLNs + false-negative SLNs). True positives were defined as an annotation by the algorithm (entirely or partly) corresponding to the area where malignant cells were detected by the pathologist, regardless of the color of its outline or size. False negatives were defined as a missing annotation by the algorithm in an area where malignant cells were detected by the pathologist. The presence of isolated tumor cells was disregarded in this study (thus missing ITC by the algorithm was not deemed as a false negative). The secondary outcome was the number of annotations made by the algorithm, as this defines the workload of the pathologist in real life. The number of annotations and corresponding confidence levels per slide were automatically extracted from the application, aggregated, and presented in an Excel spreadsheet. Inherently to selecting a subset of patients with proven nodal metastasis at diagnosis, we could not assess the false-negative rate of the algorithm in a complete population of cervical cancer patients.

Clinical, histopathological, and surgical data on the included patients were extracted from the existing database. Categorical data were summarized as frequency and percentage, and continuous variables were summarized as medians and ranges. Only descriptive statistics were performed in this proof-of-principle study. Analyses were performed using the Statistical Package for the Social Sciences version 26.0.0.1 (SPSS; International Business Machines, Armonk, NY, USA) and Microsoft Excel 2016 (Microsoft, Redmond, WA, USA). The original output of PACS and Visiopharm was used for composing the figures, leaving the annotations as posted by the pathologist or the algorithm, respectively.

## 3. Results

**Figure 3:**
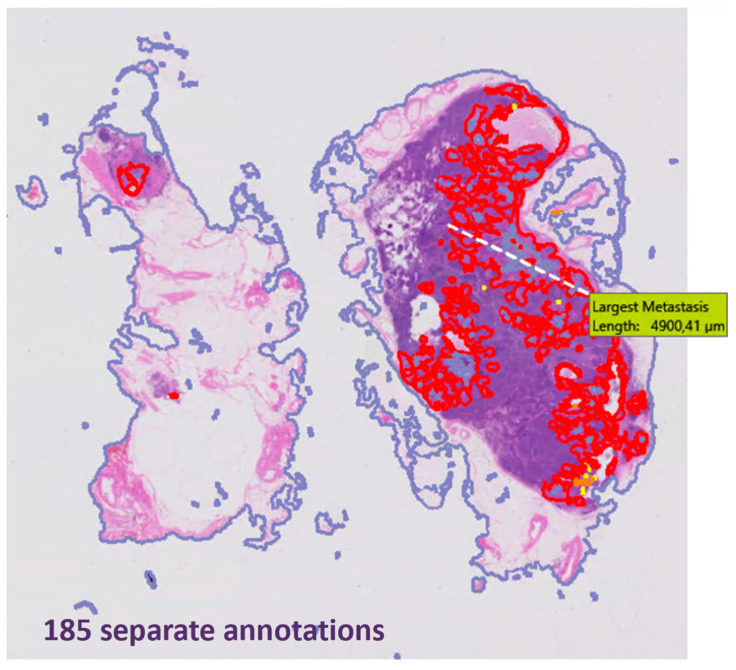
Example of algorithm output with high number of clustered annotations in a macrometastasis of squamous cell carcinoma. *As the Visiopharm application is set to European standards, the comma is used as decimal separator*.

In total, 192 patients underwent SLN procedure as part of their cervical cancer treatment during the inclusion period. Five patients objected against the reuse of their health care data for scientific research and their data were not collected. The cohort consisted of 21 patients diagnosed with early-stage cervical cancer of different histological subtypes and at least one SLN metastasis at surgical lymph node staging. Ten patients were diagnosed with macrometastasis, of which one patient was also diagnosed with micrometastasis in her bilateral SLN. Eleven patients were diagnosed with micrometastasis as their largest metastasis. Clinicopathologic features of the cohort are summarized in Table 1. In total, 47 SLNs were excised during surgery, whereof 473 H&E slides (median 18; range 6–52 slides) were reviewed by the algorithm, including frozen sections if performed. The algorithm posted 5857 annotations suspicious for metastases (all confidence levels), with a median of 128 annotations (range 32–2093) per patient. The frozen section WSIs yielded a high number of annotations probably due to the poorer section quality. When excluding frozen sections, a total of 2675 annotations were posted, with a median of 81 per patient (range 10–464), of which 1040 yellow annotations (median 39; range 7–165), 298 orange annotations (median 11; range 2–63), and 1337 red annotations (median 10; range 1–402) were posted. In slides with a high number of annotations, these were often clustered (see Figure 3).

**Figure 4:**
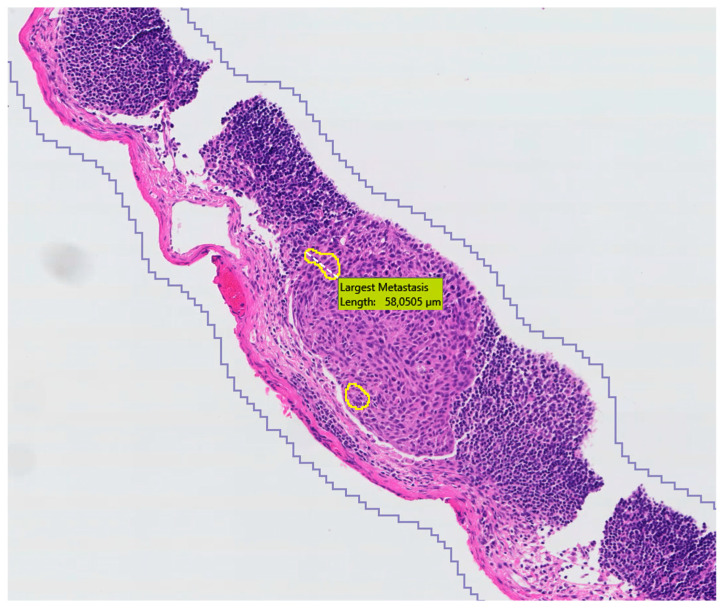
Example of algorithm output with only one yellow annotation in a larger macrometastastic area of squamous cell carcinoma. *As the Visiopharm application is set to European standards, the comma is used as decimal separator*.

On the lymph node level, the cohort consisted of 20 negative SLNs, 13 with macrometastases, and 14 with micrometastases (Table 2). In two of the 14 SLNs with micrometastases, tumor cells were only detected in the deeper-cut sections with IHC. A second pathologist reviewed these cases to confirm that the tumor cells were not visible in the H&E slides. Based on H&E slides alone, the cohort thus involved 22 negative SLNs, 13 with macrometastases and 12 with micrometastases. The algorithm picked up all H&E-positive slides with at least one annotation in the correct area, yielding a clinical sensitivity of 100%. In one case the algorithm did put an annotation in the correct area of macrometastasis but with a yellow outline only (see Figure 4).

Table 3 provides the results of the algorithm on the case level. In case 1 and case 20, tumor cells in at least one SLN were only detected in the deeper-cut IHC slides by the pathologist, indicating that the algorithm could never have picked up these tumor cells on the original H&E slides. In one case the algorithm missed an area of tumor cells on a frozen section. The regular H&E sections of this case were correctly detected by the algorithm (case 18). In one case the tumor cells were only visible in the frozen section and not in the regular H&E slides, which was correctly annotated by the algorithm (case 2). In all other cases, either no tumor was seen on the frozen sections at diagnosis or it was correctly annotated by the algorithm.

## 4. Discussion

In this proof-of-principle study, a certified AI-based algorithm, developed for adenocarcinomas of the breast and colon, showed a sensitivity of 100% for clinically relevant SLN metastasis (macrometastasis and micrometastasis) in early-stage cervical squamous cell carcinomas, adenocarcinomas, and one clear cell carcinoma. In two cases, micrometastases were only detected in the deeper-cut IHC slides and not present in the regular H&E slides, indicating that they never could have been picked up by the algorithm. The output of the algorithm amounted to a median of 128 annotations per case that had to be reviewed by the pathologist, which were not all true-positive annotations.

The confidence/color outlines, corresponding to the level of suspicion, were lumped in this study as a previous study indicated that yellow outlines may also contain tumor, necessitating review of all outlines by the pathologist [13]. Also, in the present study, orange or even yellow annotations sometimes indicated an area of metastasis. At this stage of the study, in which the algorithm is used off-label and the clinical significance of the confidence classes is to be determined, we considered all annotations of any color in an area of true metastasis as true positives.

Previous research in SLNs of breast cancer patients showed that deep learning algorithms could identify metastases in SLN slides with almost 100% sensitivity, whereas about 40% of the slides without metastases could be identified as such [16]. A challenge competition demonstrated that deep learning algorithms rival human performance. The researchers found that the best algorithm achieved similar true-positive fractions as pathologists interpreting slides without time constraints. For pathologists under time constraints, which is often the case in daily practice, the algorithms performed even better, especially in detecting micrometastases [9]. In SLNs of melanoma patients, an algorithm highlighting nodal metastases also showed promising results, accurately identifying tumor deposits >0.1 mm, without relying on immunohistochemical staining [17]. For squamous cell carcinomas, several algorithms have been employed so far, although these have not yet been implemented in clinical practice. In 2020, Pan et al. studied an algorithm developed on a slide set of metastatic esophageal squamous cell carcinoma to screen lymph nodes suspicious for metastases from the pharyngeal and lung. The applied algorithm reached an accuracy of 96.7% and 90% for pharyngeal and lung cancer, respectively [18]. In a test setting by Tang et al., nodal metastases from head and neck squamous cell carcinoma cases were detected with 100% sensitivity by an algorithm, but with less specificity (75.9%) [19].

Until recently, studies in this field were mainly undertaken on retrospective data and algorithms were sometimes even assessed as independently working entities without pathologist supervision, which is unimaginable and undesirable in a real-world clinical setting [20]. A retrospective study by Steiner et al. showed the potential of an AI-assisted workflow. Pathologists in their study considered the review of nodal micrometastases in breast cancer significantly easier when assisted by an algorithm compared to the unassisted review [11]. A recent prospective study in breast cancer using AI assistance in the clinical workflow of SLN assessment highlighted that AI-assisted pathologists not only reduced the use of IHC and costs but also felt that AI saved time and made their work more enjoyable [13].

As shown in the aforementioned prospective study, an AI-based algorithm detecting micrometastases in SLNs may obviate the need for IHC on step sections, saving up to ~€25 per slide [13]. Research in cervical cancer sentinel lymph nodes showed that routine use of IHC adds clinical value in terms of detecting micrometastases and affects the therapeutic strategy-decisions in about 1% of patients but comes with substantial costs [7]. A deep learning algorithm aiding the pathologist in adequately searching the serial H&E sections could replace the need for IHC and could thereby benefit the cost-effectiveness of pathologic ultrastaging. As also argued by Challa et al., a combined approach of algorithmic analysis followed by a pathologist review, the number of cases that require IHC can be significantly reduced [12].

Although the studied algorithm was not trained on frozen sections, our results showed that the algorithm correctly detected metastases in frozen sections in almost all cases (the algorithm missed visible tumor cells in one frozen section). In daily clinical practice, challenges arise for pathologists when reviewing frozen sections due to various artifacts affecting this tissue, such as folds and compression resulting in low image quality and the time constraints given the intraoperative consultation setting. Kim et al. proposed a method utilizing transfer learning to train deep learning models effectively for identifying metastatic breast cancer cells on WSIs of frozen sections [21]. Transfer learning involves adapting a pre-trained model from one task to another by making adjustments. The authors used annotated WSIs from frozen sections of breast cancer SLNs to train the model. Their best algorithm achieved an area under the curve of the receiver operation characteristic of 0.805 with an acceptable processing time of 10.8 min [22]. Further evaluation in clinical practice is needed, but these first results are promising for a future AI-assisted workflow, not only in conventional H&E sections but also in frozen sections.

Despite the promising perspectives of AI-assisted pathology in cancer diagnostics, several challenges remain [23]. Firstly, there is a crucial need for robust validation of AI algorithms using multi-institutional data before their clinical adoption, preferably as part of the certification procedure. This necessitates comprehensive quality control and standardization tools, as well as data sharing and validation with multi-institutional data to enhance the generalizability and reliability of these algorithms. Additionally, after implementation, continuous validation and refinement of AI algorithms by expert pathologists are imperative. Secondly, the implementation of AI assistance in clinical pathology workflows requires some hard work by information technologists and prospective studies to demonstrate the true quality and cost benefits of AI. Despite promising results from AI-based algorithms over the past five years, their integration into clinical workflows remains limited. The recent results of the CONFIDENT-B trial show the potential added value of AI assistance in daily pathology practice [24]. However, legal and ethical issues regarding the extent of pathologists’ responsibility when utilizing AI for assisted review remain unresolved. Lastly, the requirement for high-spec hardware for storage and processing all WSI output presents practical, financial, and sustainability challenges, while accreditation of the software by regulatory agencies adds another layer of complexity to the adoption of AI. Addressing all these multifaceted challenges is essential for the successful integration of an AI-assisted workflow into clinical pathology practice.

One limitation of our study is that we selected only patients with proven SLN metastasis. Thereby, the up-front probability of detecting metastases by the algorithm is high, facilitating its high sensitivity. Having perfect sensitivity means that there will be false AI alerts, substantiated by the seemingly high number of annotations we found. However, many of the annotations cluster and do not take much time to review, which was also shown in a previous breast cancer study where AI-guided review was faster [13]. Nevertheless, at present, the application used in this study works in such a way that the pathologist sees all annotations at once for a WSI, and then zooms in on the annotations separately for review. When applied in the clinical setting, however, it must remain workable for the pathologist and the display must be further optimized, e.g., by displaying annotations of the confidence classes in three galleries for rapid review. We considered all outlined areas (either red, orange, or yellow) equal as the most important within an AI-assisted workflow is that the pathologist’s attention is drawn to the area, regardless of the level of suspicion. At this stage, assessing specificity was not within the scope of our manuscript as the true-negative rate of the algorithm is considered less important than the true-positive rate, to make sure no positive cases are missed by the algorithm. Now that we have shown a proof of principle, a larger prospective study reflecting the daily practice and population is needed to accurately assess the impact of the confidence classes, assess false alerts and, more importantly, false-negative rates. Another aim of such a study would be to assess potential superior sensitivity and cost savings with an AI-assisted pathology assessment of SLN nodes. Together with the developers behind the algorithm, we should further investigate how the algorithm can be certified for use outside the targeted population and whether the confidence cut-off points for cervical carcinoma histological types might be different than those for breast and colon cancer, as well as how to cluster the annotations in one metastatic area.

## 5. Conclusions

In this proof-of-principle study, a deep learning algorithm, developed for detecting nodal metastases in adenocarcinomas of the breast and colon, performed with a 100% sensitivity for detecting SLN metastases in early-stage cervical cancer patients. No false-negative slides were observed, which shows that the application could be a useful aid to the pathologist. Our findings highlight the potential of investigating existing algorithms for off-label metastasis detection in cervical cancer and encourage prospective validation of this promising and clinically valuable application in a larger population.

## Figures and Tables

**Table 1:** Baseline characteristics on patient level.

	Patients (*n* = 21)	%
**Age, median (range)**	42 (23–63)	
**Histologic subtype**		
Squamous cell carcinoma	15	71.4
Adenocarcinoma	5	23.8
Clear cell carcinoma	1	4.8
**Histologic grade**		
Grade I	1	4.8
Grade II	8	38.1
Grade III	11	52.4
Grade not applicable *	1	4.8
**Metastasis**		
Macrometastasis	10	47.6
Micrometastasis	11	52.4
**Frozen section performed**	19	90.5
**Number of SLNs removed, median (range)**	2 (1–4)	
Number of H&E slides per patient **, median (range)	18 (6–52)	
Number of annotations per patient **, median (range)	128 (32–2093)	
**Number of annotations without frozen sections, median (range)**	81 (10–464)	
Yellow annotations	39 (7–165)	
Orange annotations	11 (2–63)	
Red annotations	10 (1–402)	

* Clear cell carcinoma; ** including frozen sections. SLN, sentinel lymph node; H&E, hematoxylin and eosin.

**Table 2:** Outcome on sentinel lymph node level.

	Sentinel Lymph Nodes (*n* = 47)
Negative *	20
**Positive**	27
**Macrometastasis**	13
*Detected with H&E* **	*13*
*Detected with algorithm*	*13*
**Micrometastasis**	14
*Detected with H&E* **	*12*
*Detected with IHC only*	*2*
*Detected with algorithm*	*12*

* Based on both H&E and IHC slides. Based on H&E slides only, 22 SLNs were negative. ** Including frozen sections. H&E, hematoxylin and eosin; IHC, immunohistochemistry.

**Table 3:** Results of the algorithm on the case level.

Case	Cancer Type	Metastasis Size	SLN Count	FS Performed	Number of Positive SLNs	Outcome of the Algorithm (Based on HE)	Visiopharm Output
**1**	Squamous	Micro	2	Yes	2	TP/**NA**	1—detected in HE+FS slides; **2—tumor cells only visible in IHC slide (deeper levels), not in H&E**
**2**	Clear cell	Macro	2	Yes	1	TP	Detected in FS slides *
**3**	Squamous	Micro	2	Yes	1	TP	Detected in H&E (not present in FS)
**4**	Squamous	Micro	2	Yes	1	TP	Detected in H&E (not present in FS)
**5**	Adeno	Macro	4	Yes	1	TP	Detected in H&E + FS slides
**6**	Adeno	Macro	2	Yes	2	TP	Both detected in H&E + FS slides
**7**	Squamous	Micro	3	Yes	1	TP	Detected in H&E slides (not present in FS)
**8**	Squamous	Macro	2	Yes	2	TP	Both detected in H&E + FS slides
**9**	Adeno	Macro	2	Yes	1	TP	Detected in H&E + FS slides
**10**	Squamous	Micro	2	Yes	1	TP	Detected in H&E + FS slides
**11**	Squamous	Micro	2	Yes	1	TP	Detected in H&E slides (not present in FS)
**12**	Squamous	Micro	2	Yes	1	TP	Detected in H&E slides (not present in FS)
**13**	Squamous	Macro + micro	2	Yes	2	TP	1—detected in H&E + FS slides (micro); 2—detected in H&E + FS slides (macro)
**14**	Squamous	Micro	2	Yes	1	TP	Detected in H&E slides
**15**	Adeno	Micro	2	Yes	2	TP	1—detected in H&E slides (not present in FS); 2—detected in H&E + FS slides
**16**	Squamous	Macro	3	Yes	1	TP	Detected in H&E slides (not present in FS) **
**17**	Adeno	Macro	4	Yes	2	TP	1—detected in H&E slides (not present in FS); 2—detected in H&E + FS slides
**18**	Squamous	Macro	2	Yes	1	TP	Detected in H&E slides, **missed in FS slides**
**19**	Squamous	Macro	2	No	1	TP	Detected in H&E slides
**20**	Squamous	Micro	1	No	1	**NA**	**Tumor cells only visible in IHC slides (deeper levels), not in H&E**
**21**	Squamous	Micro	2	Yes	1	TP	Detected in H&E + FS slides
**TOTAL**			**47**	**19**	**27**	**25**	

* No tumor cells visible in H&E slides; ** yellow annotation only. H&E, hematoxylin and eosin; FS, frozen section; IHC, immunohistochemistry; TP, true positive; NA, not applicable.

## Data Availability

The raw data supporting the conclusions of this article will be made available by the authors on request.
